# Exudative Epidermitis in Combination with Staphylococcal Pyoderma in Suckling Piglets

**DOI:** 10.3390/antibiotics10070840

**Published:** 2021-07-09

**Authors:** Lukas Schwarz, Igor Loncaric, Rene Brunthaler, Christian Knecht, Isabel Hennig-Pauka, Andrea Ladinig

**Affiliations:** 1 University Clinic for Swine, Department for Farm Animals and Veterinary Public Health, University of Veterinary Medicine Vienna, 1210 Vienna, Austria; christian.knecht@vetmeduni.ac.at (C.K.); Isabel.Hennig-Pauka@tiho-hannover.de (I.H.-P.); andrea.ladinig@vetmeduni.ac.at (A.L.); 2 Institute of Pathology, Department for Pathobiology, University of Veterinary Medicine Vienna, 1210 Vienna, Austria; igor.loncaric@vetmeduni.ac.at; 3 Institute of Microbiology, Department for Pathobiology, University of Veterinary Medicine Vienna, 1210 Vienna, Austria; rene.brunthaler@vetmeduni.ac.at; 4 Field Station for Epidemiology, University of Veterinary Medicine Hannover, Foundation, 49456 Bakum, Germany

**Keywords:** *Staphylococcus hyicus*, MRSA, epidermolysins, exfoliative toxins

## Abstract

A case of generalized exudative epidermitis (EE) is described, which occurred in a very small piglet producing farm in Austria. The antimicrobial treatment prescribed by the herd veterinarian did not improve the clinical problem. Therefore, the University Clinic for Swine intervened in the case. Lab investigations were initiated in which *Staphylococcus hyicus* (SH) and *Staphylococcus aureus* (SA), both methicillin-resistant and susceptible strains, could be isolated from the skin of affected piglets. Poor hygiene and management practices were identified as predisposing factors on site. Adaptation of antimicrobial treatment according to results of the in vitro susceptibility testing and the implementation of proper hygiene measures resolved the clinical problem. Here, we describe a fatal coinfection of SH and SA in suckling piglets.

## 1. Introduction

Staphylococci are part of the normal flora of the skin and mucosal surfaces of mammals and are considered ubiquitous in swine facilities [1,2,3]. The most common staphylococcal species causing skin disease in pigs are *Staphylococcus (S.) hyicus* (SH), the causative agent of exudative epidermitis (EE) or greasy pig disease, and *Staphylococcus aureus* (SA), which can be involved in various conditions, such as septicaemia, infection and inflammation of various tissues, including the skin in rare cases [1,4,5].

Exfoliative toxins or epidermolysins are exotoxins produced by staphylococcal species which are associated with skin lesions in humans and animals, including EE in pigs [4,6]. Epidermolysins are serine proteases that digest desmoglein-1, a calcium-dependent transmembrane glycoprotein, which is responsible for cell to cell adherence [7]. Cleavage of the extracellular part of desmoglein-1 leads to separation of cells in the stratum spinosum and exfoliation of the skin leading to the typical signs of EE. 

EE occurs worldwide usually as a sporadic problem, but occasionally major outbreaks affect large numbers of piglets and induce high piglet mortality. Piglets from 3–4 days of age may be affected and can develop a severe, generalized form of the disease. Initially, the skin turns reddish and scales develop. Those initial lesions may occur in the axillary or groin area and may remain unnoticed. Lesions develop on the skin of face and head and are first covered by serum and exudate. Later, brown to black crusts can be observed at the affected regions. Lesions can progress to cover the whole body with greasy exudate within 24–48 h [3,8,9]. Additionally, erosions and ulcers may develop in the mouth and the area of the coronary band or heel [10]. Histologically, acanthosis and formation of crusts due to hyperkeratosis and neutrophilic micro-abscesses containing numerous colonies of Gram-positive cocci can be observed. The epidermis may be ulcerated or hyperplastic, the dermis can be congested, oedematous and inflamed [3,11]. Young piglets can die within 24 h due to anorexia and dehydration. Morbidity and mortality are generally higher in younger pigs. 

Since not all cases of EE are due to SH and antimicrobial resistance is common, bacterial culture and sensitivity testing are important steps in the diagnostic work-up. Other bacteria that have isolated from pigs with EE include *Mammaliicoccus (formerly Staphylococcus) sciuri*, *S. chromogenes* and Methicillin resistant *S. aureus* (MRSA) [1]. Staphylococci can also be isolated from the skin of healthy pigs and are considered as part of the normal flora. Since predisposing factors are probably required to induce disease outbreaks, preventive measures including hygiene and housing conditions, management factors like pig flow or stocking density, and the control of other predisposing factors are of particular importance in combating EE. 

Affected pigs are treated with antimicrobial drugs. Despite treatment, piglet mortality can be high in acute outbreaks. Severely affected piglets might not recover after treatment and early treatment right after the onset of disease is crucial for success. Staphylococcal species harbour a wide variety of antimicrobial resistance genes complicating the treatment of staphylococcal associated diseases [12,13,14,15]. Antimicrobial resistance genes are often carried on mobile genetic elements, in particular plasmids and transposons, which facilitates the exchange of resistance genes not only with other staphylococci but also with other Gram-positive bacteria [15]. Since human and animal staphylococci share a large number of resistance genes [16], antimicrobial resistances of staphylococci complicate not only the treatment of affected animals but also represent a threat to humans.

The aim of this case report was to elucidate the interplay of SH with SA in suckling piglets. Furthermore, molecular biological characterisation of bacterial isolates should give important epidemiological insights into SH and SA involved in exudative epidermitis of suckling piglets.

## 2. Case Description

### 2.1. Herd Description

The case occurred on a very small, family owned farm in Austria, which is producing piglets with 25 sows in a continuous production, i.e., without batch farrowing. Piglets were sold with 30 kg of body weight. Pigs in all areas were housed on solid floor using straw bedding. Gilts were obtained from one multiplier herd. Sows produced approximately 28 total born piglets per year with an average of 23 weaned piglets per sow and year. The suckling period was at least 30 days and in some sows it was extended to a maximum of 38 days, depending on the need of farrowing pens. Due to the continuous production and lack of a batch farrowing interval, no all-in/all-out and separation of different age groups was used. Cleaning of empty rooms was performed using a pressure washer with cold water. No disinfection was performed.

Vaccination protocols included vaccination of sows against porcine parvovirus (PPV) and *Erysipelothrix rhusiopathiae* (*E. rhusiopathiae*) at the end of lactation and vaccination of piglets against *Mycoplasma hyopneumoniae* (*M. hyopneumoniae*) in the first week of life and at day 21 of life, and against porcine circovirus type 2 (PCV2) at day 21 of life. On the third day of life all piglets were supplemented with iron dextran.

Castration, teeth grinding and tail docking were performed on the second to third day of life using a non-steroidal anti-inflammatory drug prior to the zootechnical treatment.

### 2.2. Anamnesis and Physical Findings

**Figure 1:**
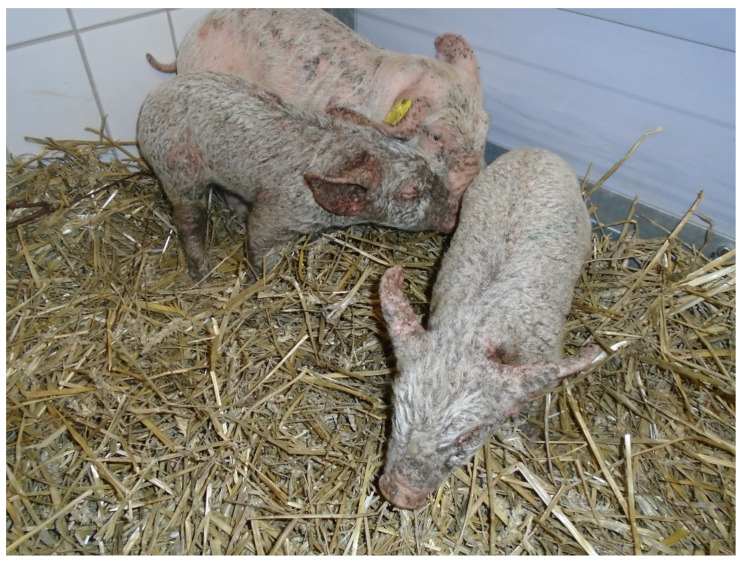
Piglets sent for further diagnostics showing typical signs of greasy pig disease.

Suckling piglets from individual litters (~2 weeks of age) started to show skin lesions resembling the typical signs of generalized greasy pig disease, including exudation, exfoliation of the skin and crusting. The herd veterinarian prescribed antimicrobial treatment with amoxicillin (15 mg/kg) via intramuscular injections to affected piglets. Since treated piglets did not show improvement of clinical signs and were wasting, with more litters becoming affected over time, the herd veterinarian consulted the University Clinic for Swine. During a following herd visit all piglets were examined for clinical symptoms. All piglets in three out of four litters showed a generalized dermatitis with typical signs of EE: exudation, crusting, pyoderma and erythema (Figure 1). Piglet mortality in the herd varied and increased from ~10% up to 25% during time periods with clinical symptoms of EE.

### 2.3. Diagnostic Methods, Laboratory and Necropsy Findings

**Figure 2:**
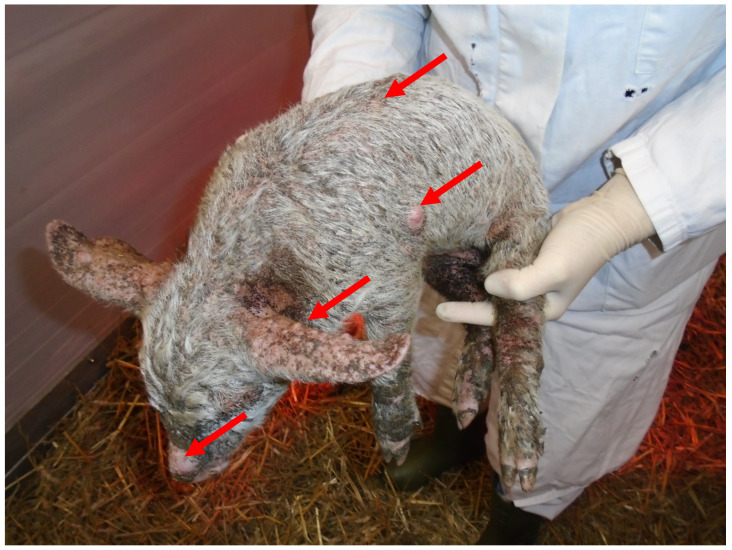
Piglet affected by exudative epidermitis and with multiple abscesses in the skin represented by red arrows.

**Figure 3:**
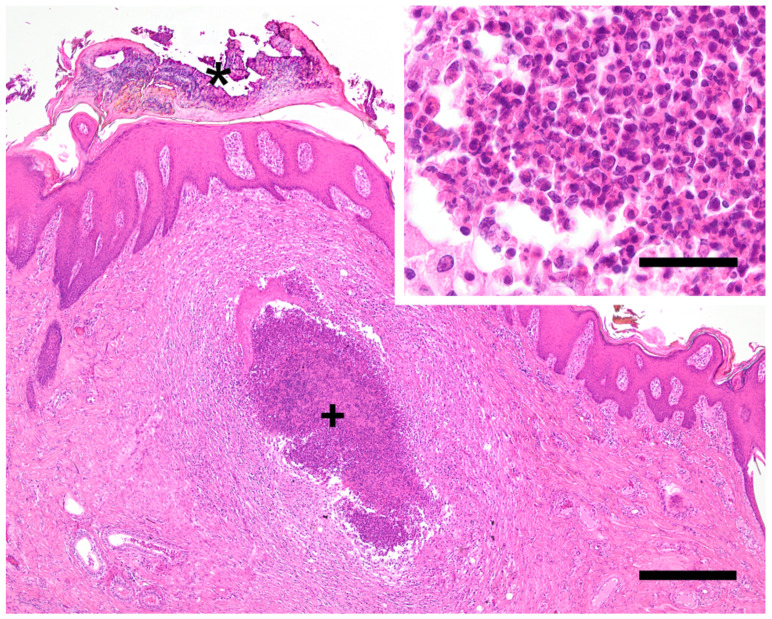
Skin of the pinna. Epidermis is covered by thick serocellular crusts consisting of keratin, neutrophils and detached epithelial cells indicated by the asterisk and intradermal abscess indicated by the cross (H&E, bar = 400 µm). Inset indicates higher magnification of pus in the superficial dermis (H&E, bar = 40 µm).

No diagnostic investigations had been initiated by the herd veterinarian prior to the farm visit conducted by the University Clinic for Swine. During the farm visit, three severely affected piglets were selected for pathological and bacteriological investigations. Pathological investigations were performed at the Institute of Pathology, Department for Pathobiology, University of Veterinary Medicine Vienna, Austria. The whole body surface of all three piglets was covered with a black, greasy, crusty layer of thickened skin (Figure 1). Additionally, multiple abscesses were diagnosed within the skin (Figure 2). Histologically, a severe purulent dermatitis with a massive accumulation of coccoid bacteria was observed (Figure 3). No further lesions were found in other organs with the exception of a purulent to abscessing lymphadenitis with accumulation of coccoid bacteria within the inguinal lymph node of one pig.

For bacteriological investigations, swabs of the wet skin lesions from each of the three piglets were taken after removing the scabs with sterile forceps. Since all piglets were pre-treated with antimicrobials, swabs of untreated animals were taken by the herd veterinarian two weeks later when newly affected piglets were present on site. All bacteriological investigations were performed at the Institute of Microbiology, Department for Pathobiology, University of Veterinary Medicine Vienna, Austria. SA was isolated from swabs of the three piglets chosen for necropsies. Both SA and SH were isolated from swabs taken from untreated piglets. Antimicrobial susceptibility testing of staphylococcal isolates was performed by agar disk diffusion according to CLSI standards (CLSI, 2020) for the following antimicrobial agents (μg/disk): penicillin (10 IU), cefoxitin (30), tetracycline (30), ciprofloxacin (5), erythromycin (15), clindamycin (2), chloramphenicol (30), gentamicin (10), rifampicin (5), linezolid (30) and trimethoprim-sulfamethoxazole (1.25 + 23.75). Results of antimicrobial susceptibility testing and associated resistance genes as well as toxin profiles are shown in Table 1. To further characterize isolated staphylococci, different molecular methods for the detection of genes encoding virulence factors were applied. SH isolate was screened for the presence of exfoliative toxins (ExhA-, ExhB-, ExhC-, ExhD-, and SHETA) by PCR [17]. SA isolates were further analysed by DNA microarray-based technology to detect over 300 different target sequences including antimicrobial resistance and virulence-associated genes, species-specific genes and SCCmec-associated genes. All isolates were genotyped by *spa*-typing and the *mecA*-positive isolates (MRSA) were further genotyped by SCCmec-associated direct repeat unit (*dru*) typing [18]. Furthermore, cefoxitin susceptible isolates (MSSA) were genotyped by multi-locus sequence typing (MLST) [19].

SH isolate (*n* = 1) was resistant to penicillin and carried exfoliative toxin B gene (*exhB*) (Table 1). The detection of *cfr* and *fexA* genes reflected the phenotypic resistance to chloramphenicol, linezolid and clindamycin and indicated *spa* type t021, sequence type (ST) 433, and clonal complex (CC) 30. MRSA isolates (piglet 1 *n* = 1; swab *n* = 1) displayed resistance to β-lactams and tetracycline and carried *blaZ*, *mecA*, and *tet*(K). MRSA isolates belonged to *spa* type t011, *dru* type dt11af and CC398. The presence of various virulence-associated genes is summarized in Table 1. Two MSSA isolates (piglet 2 *n* = 1; piglet 3 *n* = 1) had the same characteristics in common. The same applied to two MRSA isolates.

*S. aureus* isolated from one of the necropsied piglets and from the additional swabs carried methicillin resistance gene *mecA* and showed resistance to beta-lactam antimicrobials in antimicrobial susceptibility testing. Therefore, those strains were categorized as MRSA. All MRSA isolates were negative for tested toxin genes (exfoliative toxins A and B, enterotoxins, toxic shock syndrome toxin (TSST)). *S. aureus* isolates from the other two necropsied piglets did not carry the *mecA* gene (methicillin susceptible *S. aureus*, MSSA) but were positive for enterotoxin G and TSST. *S. hyicus* isolates were positive for exfoliative toxin B.

### 2.4. Further Steps and Outcome of Case

Antimicrobial treatment of affected piglets was adapted according to results of the in vitro susceptibility testing of all isolated staphylococci, i.e., SH, MRSA and MSSA. As soon as the first skin lesions were observed in suckling piglets, all piglets within the litter were treated with long acting marbofloxacin (8 mg/kg, Forcyl, Vetoquinol Österreich GmbH, Vienna, Austria) intramuscularly. In addition, the farmer was advised to improve the hygiene on his farm. A proper cleaning and disinfection protocol was set up, including a power wash with hot water and detergents to dissolve the biofilm followed by drying and disinfection. Since mycotoxin analyses of feed showed high contamination with deoxynivalenol (DON 7050 µg/kg), the feed ratio was changed, i.e., corn was reduced in order to reduce the mycotoxin content administered to pigs. The clinical problem resolved after implementing proper hygiene measures and changing the antimicrobial treatment of affected piglets.

## 3. Discussion

The present report describes a case of generalized EE in suckling piglets in a small piglet producing farm in Austria. During the farm visit, several predisposing factors for EE were observed, including poor hygiene and management protocols, such as the lack of proper cleaning and disinfection and no all-in/all-out and mixing of different age groups. The role of high mycotoxin levels as a predisposing factor in the present case is not clear but it could be speculated that immunosuppressive effects of mycotoxins [20] might have predisposed piglets to the development of the disease.

The herd veterinarian prescribed treatment with amoxicillin after first signs of dermatitis had occurred in suckling piglets but no bacterial isolation and antimicrobial susceptibility testing was initiated even when antimicrobials did not improve the situation. Since antimicrobial resistances are common in staphylococci, treatment without lab investigation is considered critical. Suggesting an involvement of staphylococci and streptococci in diseased animals, antimicrobial treatment should always be performed after susceptibility testing. Lab investigations initiated after the farm visit of the University Clinic for Swine showed that the treatment with amoxicillin might have been effective against some of the isolated staphylococci but not against others. It can be speculated that the antimicrobial treatment might have been favourable for the induction of antimicrobial resistances on the farm, since it has been shown that antimicrobial resistance patterns are influenced by the use of antimicrobials in pigs [21]. Particularly for treatment of bacteria showing a wide variation of antimicrobial resistances, a judicious use of antimicrobials is essential. One of those pathogens, which has received considerable interest over recent years and was also isolated in the present case herd, is MRSA. 

Hospital associated MRSA (HA-MRSA) have emerged since the late 1960s and nosocomial infections have become more frequent since the 1990s [22,23]. Livestock associated MRSA (LA-MRSA) have been intensely investigated in pigs ever since the detection of widespread nasal colonization of pigs and pig farmers in the Netherlands [24,25]. Nasal colonization of pigs by LA-MRSA has been shown in several countries including France [26], Belgium [27], Denmark [28], Germany [29,30], but also Canada [31], the US [32] and Singapore [33]. MRSA belonging to CC398 is the predominant LA-MRSA clone in Europe. Those ST398 strains can be transmitted to humans [31]; however, they are more adapted for colonizing pigs. It has been shown that people in direct contact to pigs are at greater risk of being carriers of LA-MRSA in the nasal cavity [22]. The presence of MRSA in farmers has been shown to be strongly related to the duration of animal contact. The prevalence of MRSA in the nasal cavities during absence of animal contact was rapidly decreasing with only few individuals appearing to be persistent carriers [34]. Therefore, contamination of the nasal cavity might be more frequent than real colonization and LA-MRSA can be seen as a poor persistent colonizer in humans compared to HA-MRSA [35,36,37,38]. Moreover, human to human transmission of LA-MRSA seems to be less frequent compared to HA-MRSA [22,39]. Nevertheless, LA-MRSA is able to cause infections in humans; for people at risk, it is important to know if they are carriers of LA-MRSA when entering hospital, in particular when surgery is planned. For this reason, some countries like the Netherlands, screen farmers for the presence of LA-MRSA when entering hospital in order to minimize infection with LA-MRSA in humans. So far, in Austria, MRSA belonging to CC398 lineage has been isolated from humans [18], diverse companion animals [18,40], ruminants and New World camelids [41,42], but there is no published information on the presence of this clone in the Austrian pig population. There is solely information regarding the presence of CC398 in the dust samples of pig breeding facilities from 2006 [43]. Therefore, most likely, the CC398 has been present in the Austrian pig population for more than a decade. In general, the information on the presence of toxigenic *Staphylococcus hyicus* is scarce. SH carrying *exhB* of porcine origin have been isolated from different European countries, including Denmark, Belgium, Croatia and Germany as well as the USA [44,45].

CC30 is a common human lineage [46,47,48,49] and has been identified as a carrier of bovine leukocidin *lukM/lukF-PV (P83)* in isolates of bovine origin [50]. It is assumed that this leukocidin plays an important role in the etiology of bovine mastitis. The LukM/LukF-PV(P83) leukocidin only kills bovine neutrophils [51,52]. Nevertheless, this leukocidin has also been detected in MRSA belonging to CC30 of porcine origin [53]. Therefore, this lineage may be of concern for porcine health. Interestingly, the *lukM/lukF-PV (P83)* gene was detected in a CC30 strain originating from an Austrian marmot [54]. Just like the strain from marmot, two CC30 strains harbor multidrug resistance gene *cfr. cfr* confers resistance to all phenicols, lincosamides, oxazolidinones, pleuromutilins, and streptogramin A. Due to the importance of these classes of antimicrobials, especially oxazolidinones, in the therapy of staphylococcal infections in human medicine, the detection of MSSA carrying this gene may be of public health concern. 

Even though Austria has a large population of domesticated pigs and highly developed production, there is no report on the presence of SH carrying exfoliative toxin genes in the Austrian pig population. Thus, the present study describes the presence of exotoxins involved in exfoliative activity for the first time in Austrian pigs. 

The farmer and his family were advised to request testing for MRSA prior to any surgery or submission to a hospital.

## 4. Conclusions

The exact role of the MRSA in the clinical signs of EE in affected piglets of the present case is not completely clear. The involvement of MRSA in EE has been previously described [5,13]. In the present case, both SH and SA could be isolated from EE affected piglets. Since all isolated SA strains were negative for epidermolysins, it can be speculated that primary lesions of EE were initiated by SH. *S. aureus* could have been a secondary invader, which maintained skin lesions after SH has been eliminated by treatment with amoxicillin. By all means, initiated antimicrobial therapy was effective against both agents.

## Figures and Tables

**Table 1:** Summarized main molecular characterization, antimicrobial resistance and toxins profile of the *Staphylococcus* isolates investigated.

**Isolates**	**Species ^1^**	**CC ^2^**	**ST ^3^**	***spa***	***dru***	**Antimicrobial Resistance Profile**		
						**Phenotype ^4^**	**Genes Detected**	
Piglet 1, Swab	MRSA	CC398	n.t.	t011	dt11af	β-lactams, TET	*blaZ*, *mecA*, *tet*(K)	
Piglet 2, Piglet 3	MSSA	CC30	ST433	t021	n.a.	CHL, LZD, CLI	*cfr*, *fexA*, *fosB*	
Swab	SH	n.a.	n.a.	n.a	n.a.	PEN	n.a.	
**Isolates**	***cap*** **Gene (*cap* 8)**	***cap*** **Gene (*cap* 5)**	**Hemolysins**	**Leukocidins (Luk) Components**	**Biofilm-Associated Genes**	**Adhesion Factors**	**Exfoliative Toxins**	**Enterotoxins and Enterotoxin-Like Genes**
Piglet 1, Swab	NEG ^5^	POS ^5^	*hla*, *hlb*, *hld*, *hlgA*	*lukF*, *lukS*, *lukX*, *lukY*	*icaA*, *icaC*, *icaD*	*clfA*, *clfB*, *cna*, *fnbA*, *fnbB*		
Piglet 2, Piglet 3	NEG	POS	*hla*, *hlb*, *hld*, *hlgA*	*lukF*, *lukS*, *lukM/lukF-PV* (*P83*), *lukX*	*icaA*, *icaC*, *icaD*	*clfA*, *clfB*, *cna*, *fnbA*, *fnbB*		*egc*, *sei*, *sem*, *sen*, *seo*, *seu*
Swab	n.a.	n.a.	n.a.	n.a.	n.a.	n.a.	*exhB*	n.a.

Legend Table 1: ^1^ S = Species, MRSA = methicillin-resistant *Staphylococcus aureus*; MSSA = methicillin-susceptible *Staphylococcus aureus*; SH = *Staphylococcus hyicus*; ^2^ clonal complex; ^3^ sequence type; ^4^ Phenotype: PEN = penicillin; CHL = chloramphenicol; LZD = linezolid, CLI = clindamycin; TET = tetracycline; ^5^ NEG = negative, POS = positive, n.t. = not typable, n.a. = not assessed; AMR = antimicrobial resistance.

## Data Availability

All data of this case report is presented in the manuscript.
